# Towards laser printing of magnetocaloric structures by inducing a magnetic phase transition in iron-rhodium nanoparticles

**DOI:** 10.1038/s41598-021-92760-5

**Published:** 2021-07-02

**Authors:** Ruksan Nadarajah, Joachim Landers, Soma Salamon, David Koch, Shabbir Tahir, Carlos Doñate-Buendía, Benjamin Zingsem, Rafal E. Dunin-Borkowski, Wolfgang Donner, Michael Farle, Heiko Wende, Bilal Gökce

**Affiliations:** 1 Universitätsstr. 7, 45141 Essen, Germany; 2 Faculty of Physics and Center for Nanointegration Duisburg-Essen (CENIDE), University of Duisburg-Essen, Lotharstr. 1, 47057 Duisburg, Germany; 3 Institute of Materials Science, Technical University of Darmstadt, Alarich-Weiss-Strasse 2, 64287 Darmstadt, Germany; 4 Materials Science and Additive Manufacturing, University of Wuppertal, Gaußstr. 20, 42119 Wuppertal, Germany; 5 Ernst Ruska-Centre for Microscopy and Spectroscopy With Electrons and Peter Grünberg Institute, Forschungszentrum Jülich GmbH, 52425 Jülich, Germany

**Keywords:** Nanoscale materials, Magnetic properties and materials, Lasers, LEDs and light sources

## Abstract

The development of magnetocaloric materials represents an approach to enable efficient and environmentally friendly refrigeration. It is envisioned as a key technology to reduce CO_2_ emissions of air conditioning and cooling systems. Fe-Rh has been shown to be one of the best-suited materials in terms of heat exchange per material volume. However, the Fe-Rh magnetocaloric response depends on its composition. Hence, the adaptation of material processing routes that preserve the Fe-Rh magnetocaloric response in the generated structures is a fundamental step towards the industrial development of this cooling technology. To address this challenge, the temperature-dependent properties of laser synthesized Fe-Rh nanoparticles and the laser printing of Fe-Rh nanoparticle inks are studied to generate 2D magnetocaloric structures that are potentially interesting for applications such as waste heat management of compact electrical appliances or thermal diodes, switches, and printable magnetocaloric media. The magnetization and temperature dependence of the ink’s γ-FeRh to B2-FeRh magnetic transition is analyzed throughout the complete process, finding a linear increase of the magnetization M (0.8 T, 300 K) up to 96 Am^2^/kg with ca. 90% of the γ-FeRh being transformed permanently into the B2-phase. In 2D structures, magnetization values of M (0.8 T, 300 K) ≈ 11 Am^2^/kg could be reached by laser sintering, yielding partial conversion to the B2-phase equivalent to long-time heating temperature of app. 600 K, via this treatment. Thus, the proposed procedure constitutes a robust route to achieve the generation of magnetocaloric structures.

## Introduction

2D-laser printing has gathered increasing interest for the fabrication of large-scale flexible and opto-electronic elements such as printed circuit boards^1,2^, organic light-emitting diodes (OLEDs)^3,4^, solar cells^5,6^ and supercapacitors^7,8^. Nanoparticle dispersions such as silver inks are commonly used to produce 2D conductive paths for electronics^9,10^ and sensor technology^11,12^. The formation of 2D structures from nanoparticle inks can be achieved by localized heating by laser irradiation leading to ultrafast sintering and rapid cooling of the particles, which is not possible using conventional oven sintering. 2D-laser sintering has been used to fabricate circuits with intricate micro-patterns possessing high electrical conductivity^13^. Compared to conventional sintering approaches, laser sintering provides rapid processing speed^14–16^ and enables damage-free fabrication^17,18^, better design selectivity^19,20^, and process control^21–23^.

2D printing has been vastly explored and adapted for electronic applications. However, the printing of magnetic nanomaterials has been barely explored^24^. Especially among magnetic materials, magnetocaloric materials have gained a notable interest due to their potential for replacing conventional gas compression-based refrigeration systems^25^. The magnetocalorically mediated cooling mechanism is an environmentally friendly process that does not require polluting refrigerants. It has been reported that more than 1000 tons of environmentally harmful hydrofluorocarbons (HFC) are produced for cooling appliances per year, and these have a global warming potential of 1000 to 3000 times that of CO_2_ emissions^26^. In addition, according to the international energy agency (IEA) report, 20% of the total household energy consumption is employed in cooling appliances^27^. It is predicted that if the energy conversion efficiency of cooling systems is not improved, the electricity consumption for these appliances will triple by the year 2050^28^. Magnetocaloric materials can replace conventional refrigerants, consequently the development of these materials is interesting, not only to eradicate the use of greenhouse gases but also to provide a higher cooling efficiency than conventional systems^29^. This is proven by the fact that until now the magnetic-based cooling system has already demonstrated 20% higher Carnot efficiency compared to compress gas refrigeration systems^26^. These materials rely on the principle of the magnetocaloric effect (MCE); thereby exhibiting an adiabatic change in temperature in response to the application of a magnetic field^30^. Brown et al.^31^ have demonstrated the magnetocaloric effect of Gadolinium (Gd); producing a gigantic cooling effect around 294 K; a milestone towards the magnetic cooling near room temperature. A handful of research has explored different materials for magnetocaloric applications in the last few decades. The giant magnetocaloric effect (GMCE) was observed in La(Fe, Mn, Co, Mn)_13-x_Si_x_(H,N, C)_y_, MnAs_1-x_Sb_x_, Fe_2_P-types compounds (MnFeP_1-x_As_x_), Ni-Mn-based Heusler and La_1-x_Ca_x_MnO_3_ manganites^31^. Also, other ferromagnetic perovskites, glass ceramics, oxide based composites and spinel ferrites exhibit magnetocaloric effect^32^.

Among magnetocaloric materials, the nearly equiatomic FeRh alloy is one of the most promising materials, as it exhibits the largest magnetocaloric effect in terms of adiabatic temperature change^33^. Iron-rhodium with a composition near the equimolar ratio undergoes a first-order as well as a second-order phase transition during heating^34^. At room temperature, the FeRh system is in the B2-phase with an antiferromagnetic (AFM) order. At around T_pt_ = 360 K, depending on the composition, the first transition from antiferromagnetic (AFM) to ferromagnetic (FM), accompanied by a 1% volume expansion, is observed upon applying a magnetic field^35,36^. The magnetic moment of each atom measured by neutron diffraction experiments revealed that in the AFM phase, each Fe atom has six adjacent Fe atoms with spins in the reverse direction. Each of them carries a magnetic moment of µ_Fe_ = 3.3 µB, while the Rh atoms are not magnetic. In the FM state, Fe carries a magnetic moment µ_Fe_ = 3.2 µ_B_, while the Rh also carries a magnetic moment of µ_Rh_ = 0.9 µ_B_^37^. The magnetic properties of Fe-Rh alloy systems were first studied by Fallot in 1938, he found out that when the temperature increases to a critical value, there is a sudden increase in magnetization^38^. This was measured later by Kouvel and Hartelius in 1962. It was found that by the application of a fixed magnetic field of 5kOe at a range of temperatures, a sharp transition was observed at 350 K with a saturation magnetization of 130 emu·g^-1^, while at higher temperatures FeRh was ferromagnetic^38^. FeRh, as a material class for refrigeration, was first investigated by Annaorazov et al^39^ in 1992. The temperature and entropy change for the Fe_49_Rh_51_ alloy system at different applied magnetic fields and temperature regimes of annealed and quenched samples were measured. Their results showed that the largest magnetocaloric effect was witnessed at the applied field of 2 T and a temperature of 308.2 K, where a temperature drop of 12.9 K and refrigerant capacity of 135.22 J kg^-1^ T^-1^ was observed for the quenched sample. FeRh near to equimolar composition nowadays possesses one of the highest refrigerant capacities even surpassing Gd_5_Si_2_Ge_1.9_F_0.1_ and MnFeP_0.45_As_0.55_ alloys^40^. FeRh’s second-order transition takes place at the Curie temperature of T_C_ = 650 K, where the phase changes from the ferromagnetic B2-phase to the γ-phase which is paramagnetic (PM). This transition makes this system interesting for heat-assisted magnetic recording^41,42^, cooling systems^43,44^, and (bio-)hyperthermia^45,46^. Although FeRh is an expensive material, the use of this material for nanoscale or microscale applications reduces the material amount required making it an attractive option due to its superior performance.

The references discussed are restricted to the alloy in the bulk regime. As the particle size is reduced to the nanoscale, properties such as melting point, electrical conductivity, chemical reactivity, and magnetic permeability change. This is due to the increase in surface-to-volume ratio and quantum confinement effects. Although FeRh nanoparticles have not been extensively studied, in most cases, their properties were quite different from the bulk material, depending on the synthesis method. Smekhova et al^47^ synthesized Fe_50_Rh_50_ nanoparticles using a hydrogenation process with subsequent characterization by X-ray magnetic dichroism (XMCD), showing that the magnetic moment of Fe is reduced by a factor 2 compared to bulk. Also, Amiens et al^37^ synthesized FeRh alloy nanoparticles using simultaneous decomposition of Fe and Rh precursor and found out that the magnetization of the Fe atoms was less than half compared to the bulk alloy of similar composition characterized by SQUID magnetometry. In some cases, the wet chemical process results in non-magnetic FeRh nanoparticles with nearly zero coercivity due to the formation of a disordered FCC structure, while the coercivity increases after annealing^48^. Due to their large particle size to volume ratio, these particles are prone to oxidation. The oxidation not only leads to a change in magnetic properties, but it also affects the stoichiometry of Fe and Rh, leading to inhomogeneous composition and thereby indirectly affecting the magnetic properties as well. Previously^49^, we developed a method to produce nearly oxygen-free FeRh nanoparticles using laser ablation of an FeRh target in organic liquids, where a broad range of number-weighted particle diameters ranging from 5 to 100 nm, with about 5 at. % oxidation of the alloy nanoparticles could be obtained. This process has two main advantages; first, the synthesis process only involves the FeRh target and the selected liquid, generating ligand-free nanoparticles and avoiding byproducts that can influence the physical, chemical, electrical and magnetic properties of the alloy nanoparticles. The influence of the ligands was recently demonstrated by Trindade et al^50^, showing that the anisotropic electrical conductivity of printed flexible metal parts was mainly due to the organic molecules present in the ink. Second, the broad particle size distribution will facilitate pore-free sintering of FeRh nanoparticles. This was also observed by Ma et al.^51^ in that work a broad particle size distribution enhances the overall sintering features increasing the packing density and reducing the isolated pores.

Although the magnetocaloric properties of a material are the crucial criteria for magnetocaloric cooling applications, the design of the system is also essential for efficient device performance. The development of a magnetic cooling system requires geometries that can enable quick and efficient heat transfer to the transfer fluid^52^. Also, it demands high mechanical stability of the magnetocaloric structure to undergo thousands of refrigeration cycles. Since some magnetocaloric materials are based on rare-earth metal elements, with the associated limitations due to their low abundance, alternative magnetocaloric materials are required for the sustainable development of this cooling technology. Besides, a proper design is needed to get maximum efficiency with minimum material usage. 2D-laser printing has been proven to be a versatile technique to fabricate custom structures, reducing material usage and achieving mechanically stable structures^53^. This technique can be used for printing magnetocaloric materials for microcooling systems, thermal switches, pumps for microfluidic applications and energy harvesting devices, thereby precisely controlling the printing parameters and achieving complex geometries according to the requirement^40^. For example, in the semiconductor industry, the number of transistors in an integrated circuit increases every year. The shrinkage in size leads to an increase in current densities. Heat dissipation leads to the melting of such transistors; therefore, the technology demands better heat management for proper heat dissipation. Systems such as mechanical fans and solid-state thermoelectric refrigerators can only dissipate a small amount of heat due to the low coefficient of performance (COP). Therefore, an effective alternative to such devices are magnetocaloric micro cooling prototypes, which have demonstrated an efficient cooling mechanism and easy manipulation and control of the generated structures^54^.

Consequently, 2D printing of magnetocaloric nanoparticle inks is aimed not only to facilitate the building of solid-state refrigeration devices of complex geometries but also to reduce material usage, increase heat transfer efficiency and enhance the magnetocaloric performance by improved nanoparticles sintering characteristics (compositional homogeneity, reduction in porosity, densification, and grain growth).

## Experimental

FeRh nanoparticle colloids were synthesized by the method of laser ablation in liquid (LAL)^55,56^, which is a method used in recent years in the context of laser printing^57–59^. The synthesis was carried out in acetone with an argon atmosphere using a picosecond-pulsed Nd:YAG laser (Ekspla, Atlantic Series, 10 ps, 100 kHz, 80 μJ, 1064 nm). The ablation target was custom-made by the **"**Research Institute for Noble Metals and Metal Chemistry**"**, Schwaebisch Gmuend, Germany and has an equimolar FeRh composition. An incident laser fluence of 3.5 J/cm^2^ with a Gaussian beam profile was employed to irradiate the target. More information on the LAL setup can be found elsewhere^49,60^. Temperature-dependent x-ray diffraction (XRD), magnetometry, and Mössbauer spectroscopy analyses were performed to observe and characterize the phase transition of the LAL synthesized FeRh nanoparticles. Phase analysis was conducted by XRD using Mo K-alpha radiation in a transmission geometry on a custom-built setup with a Mythen2 R 1 K detector (Dectris Ltd). The sample was mixed with NIST 640d standard silicon powder to correct geometric errors and glued on a graphite foil with high-temperature varnish. The temperature was controlled via a closed cycle He-cryostat (SHI Cryogenics Group) up to 700 K. Further phase analysis and magnetic characterization was performed using Mössbauer spectroscopy and magnetometry measurements. For the latter, the colloidal particles were dried under argon flow, and the nanoparticle powder was pressed into a small pellet (ca. 3 × 2 mm, ca. 10 mg), which was mounted on the heater stick of the vibrating-sample magnetometer (VSM) option of a Quantum Design PPMS DynaCool. After recording M(H)- and M(T)-curves of the sample’s original state, the sample was heated in steps of 50 K up to 973 K to check the magnetization dependence with the temperature that can be related to the phase transition from γ-FeRh to B2-FeRh, which is expected to take place during the heating process by laser printing. After a maximum annealing temperature of 973 K is reached, magnetocaloric experiments were performed on the annealed pellet, recording M(H)-curves in a temperature range of 5 – 400 K, and checking for the presence of the field-induced AFM-FM phase transition. After each M(H)-sweep, the sample material was re-initialized to the completely FM state, by heating above the phase transition, followed by cooling to 5 K, before approaching the next temperature point. Mössbauer spectra were acquired in transmission geometry using the constant acceleration mode. To record spectra in the 30—500 K temperature range, a liquid Helium (l-He) cryostat and a vacuum-oven were employed. The measurements were performed on the resulting FeRh-nanoparticles after annealing during the magnetometry experiments; the dried powder was mixed with chemically inert boron nitride and pressed to a cylindrical disc for Mössbauer experiments. Furthermore, temperature-dependent transmission electron microscopy (TEM) analysis (JEOL 2200FS) was performed to investigate the phase segregation effects. To control the sample temperature during TEM measurements, it was dropcasted on a SiN Microelectromechanical systems (MEMS) chip and placed in a Protochips Aduro heater holder to set up high in-situ temperatures using a heating resistor. To perform the laser sintering of the generated nanoparticles, an FeRh ink was formulated with a colloidal FeRh nanoparticle concentration of 1 wt%. A 25 × 37 cm glass substrate was coated with 100 µL of 10 wt% Polyvinylpyrrolidone (PVP)-solution in ethanol. Afterward 200 µl of the ink was drop cast on a PVP-coated glass substrate. PVP acts as an adhesive between the FeRh nanoparticles and the substrate and it also assists the particle consolidation during the sintering process^61^. The obtained film was then sintered using a continuous wave (CW)-laser (Laser Quantum, 532 nm) and a 2-axis linear stage (Thorlabs DDSM100/M). The influence of the laser and scanning parameters during sintering was evaluated. A densely sintered structure was achieved at a scanning speed of 10 mm/s, a sixfold repetition of the irradiation pattern, and a laser intensity of 0.85 W/mm^2^. At lower intensities, no or very few nanoparticles were sintered, while substrate damage was observed at higher intensities. The laser sintering was confirmed by rinsing the substrate with ethanol, the sintered lines remained intact on the substrate, and the unsintered ink was removed. We further performed TEM analysis to investigate the effect of laser sintering on the surface morphology and phase transformation of the FeRh particles. For this, laser sintering was directly performed on an electron transparent 30 nm SiN membrane which contained the FeRh particles. The membrane was evaporated during the sintering process. The sintered FeRh, however, remained as free-standing flakes that were suitable for subsequent TEM analysis. Bright-field and HR-TEM measurements were performed on this flake to investigate the structure and morphology of the laser-sintered structures.

## Results and discussion

### Temperature dependence

**Figure 1:**
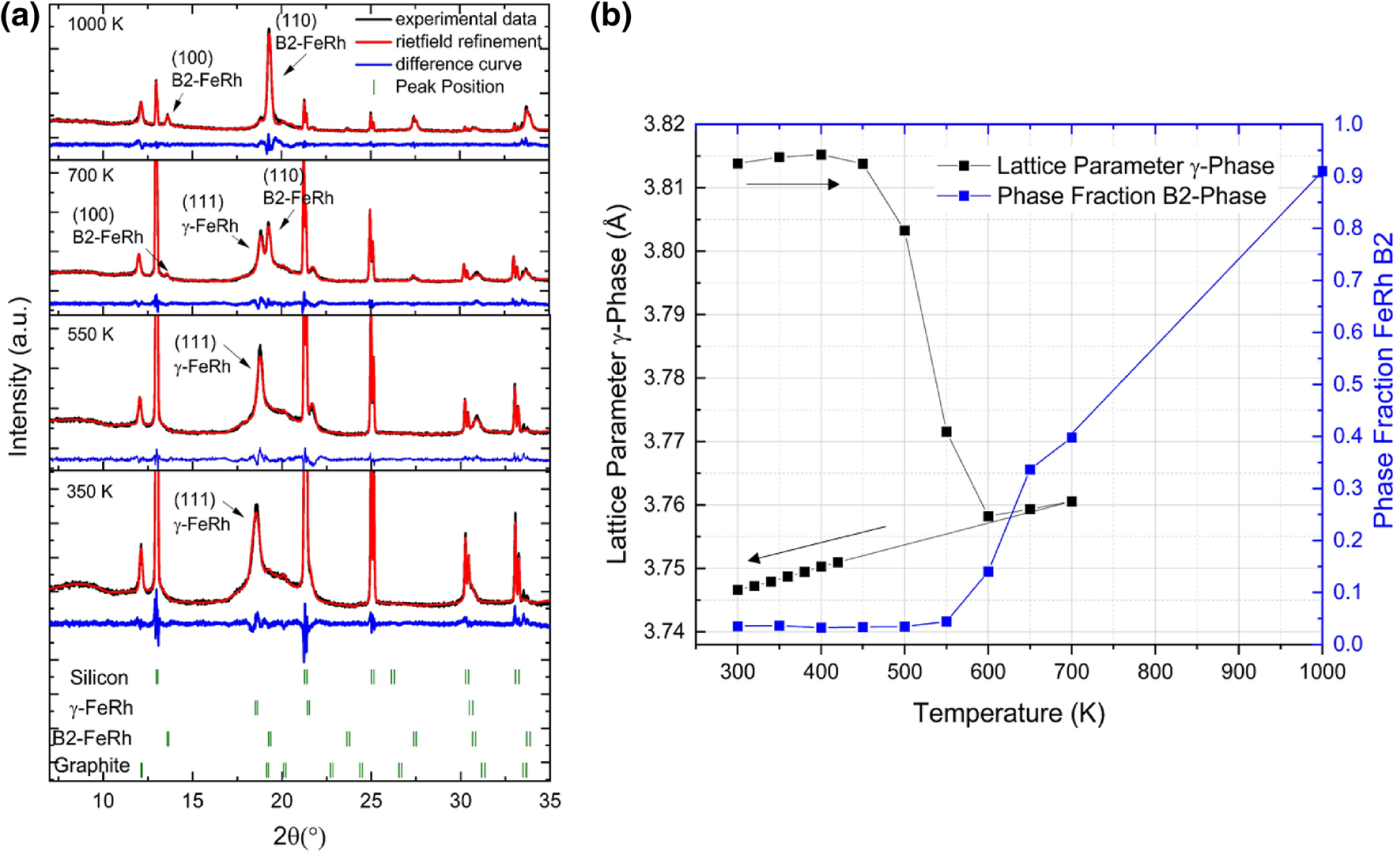
(**a**) XRD data of FeRh nanoparticles obtained by laser ablation in acetone after annealing in situ at 350 K, 550 K, 700 K. For 1000 K, the sample was heated in a furnace and measured afterwards at 300 K. The peak positions of silicon, γ-FeRh, B2-FeRh and graphite are indicated. (**b**) Dependence of the lattice parameter for the γ-phase and the phase fraction of the B2-phase with the temperature.

The temperature dependence of the phase transition of the laser synthesized Fe_50_Rh_50_ nanoparticles are evaluated by XRD, magnetometry and Mössbauer spectroscopy. Figure 1 shows the XRD patterns of the FeRh nanoparticles as a function of the temperature. At ambient temperature, the crystalline sample fraction consists of the γ-FeRh phase, as visible from the (111)-reflection with strong diffraction peaks at 18.5°, 21.4° and 30.4°. With increasing temperature, a reduction of the γ-phase, and an increase of the B2-phase is observed. Rietveld refinement with “Fullprof” software was used to analyze the lattice parameter and the change of the phase fraction^62^. It can be observed that at 350 K, around 97% of the crystalline sample fraction is in the γ-phase and only 3% in the B2-phase. Starting at 600 K, a linear increase of the (110)-peak is observed, indicating the formation of the B2-phase. After annealing at 1000 K in a furnace and water quenching, 90% of the crystalline sample is in the B2-phase. Further studies were performed to quantify the change in the phase fraction for lower temperatures and longer annealing times. In Figure S1 it can be observed that even though the B2-fraction increases with time, it does so by no more than 5% after 5 h of annealing at 700 K. This leads to the conclusion that temperature values of 1000 K or higher are needed to transform (almost) the whole sample into the B2-phase. Furthermore, a change of the γ-phase lattice-parameter was observed at about 500 K, decreasing from 3.814 Å to 3.747 Å. It is hypothesized that this lattice constant reduction of app. 2% can be explained by the temperature-induced removal of hydrogen from the lattice, which could have been incorporated during the synthesis due to the presence of acetone, a hydrogen-containing solution, explaining the higher original lattice parameter.

**Figure 2:**
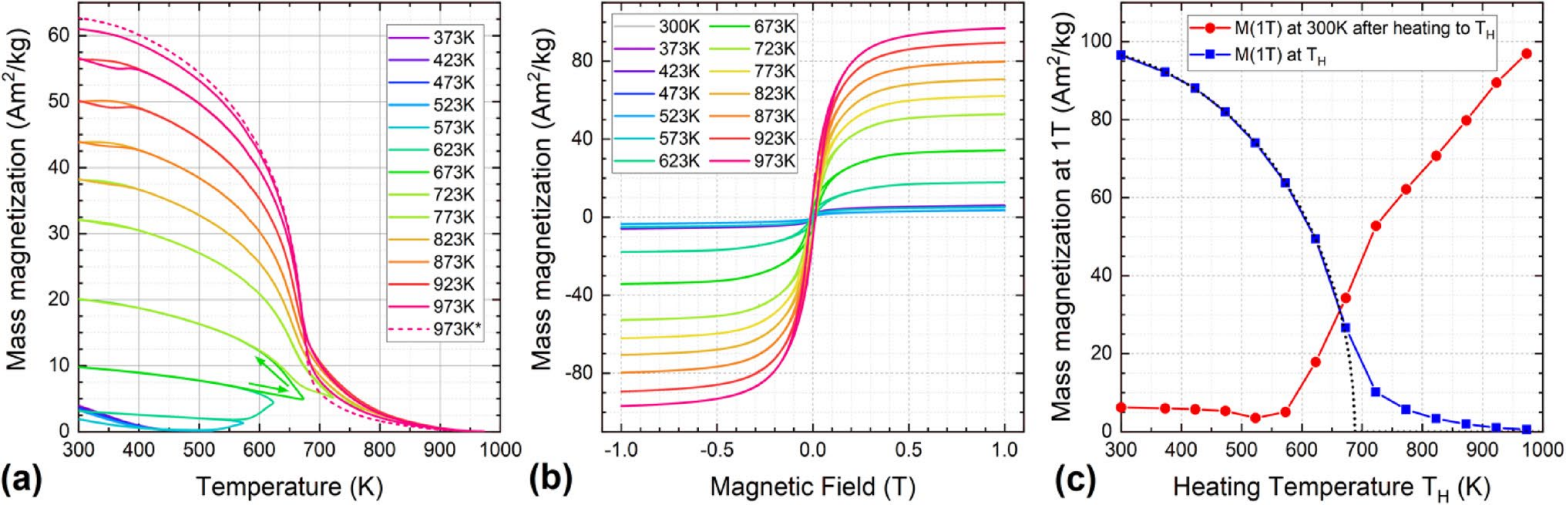
(**a**) Subsequent M(T)-curves were recorded at 0.1 T with increasing annealing temperature T_H_; the last temperature cycle was measured twice (dashed) to check reproducibility (**b**) M(H)-curves of FeRh nanoparticles recorded at 300 K after annealing at different temperatures T_H_, (**c**) magnetization values measured at 1 T as a function of T_H_ (blue, see Fig. S2) interpolated via the Brillouin function (dotted, for details see text) and measured at 300 K after each annealing step (red, from (**b**)).

To study the phase transition to the B2-state induced by annealing at different temperatures in further detail, the following procedure was utilized. First, the temperature-dependent magnetization M(T) was recorded at a sweep rate of 2 K/min, and an applied magnetic field of 0.1 T (Fig. 2a) while the FeRh-nanoparticle pellet was heated in steps of 50 K to evaluate the effect of different annealing temperatures T_H_. After each heating and cooling cycle, the sample temperature was fixed to 300 K and M(H) curves up to maximum fields of 1.0 T were recorded, in order to analyze the irreversible increase in magnetization as a result of the transition of γ-FeRh into the B2-phase (Fig. 2b). While raising the annealing temperature, a first irreversible increase in M(T) is visible at around 550 K, with a maximum increase per cycle of the room temperature magnetization being induced at approximately 700 K. The maximum annealing temperature, 973 K, leads to a magnetization M(0.1 T, 300 K) of app. 62 Am^2^/kg. Looking at the trend in magnetization measured at 300 K after annealing, as shown in Fig. 2 (c, red), an almost linear increase is observed starting at ca. 550 K, closely resembling the results from XRD showing the B2-fraction increase with temperature, Fig. 1 (b, blue). This fact indicates that the rising magnetization is directly linked to the B2-phase content (Fig. S4). Checking in detail the 300—450 K temperature range in Fig. 2a, the first indications of the reversible thermal hysteresis assigned to the AFM-FM transition characteristic for B2-FeRh are present, starting at app. T_H_ ≈ 773 K, and increasing in intensity up to the maximum annealing temperature. An additional curve at 973 K (dashed line) was measured to check the reproducibility, repeating the annealing to 973 K and cooling to 300 K. M(T)-curves corresponding to the highest values of T_H_ also show a decrease in magnetization above the Curie temperature of FeRh, which is typically approximately 675 K^63^. This could originate from the decomposition of a minor oxide contribution present in the original sample material with a higher ordering temperature. To compare the effect of a higher magnetic field, 1 T-M(H) curves were also recorded at the respective annealing temperatures T_H_ to characterize the temperature-dependent high-field magnetization (Fig. S2). The results are shown in Fig. 2 (c, blue), allowing us to estimate the Curie temperature to be ca. 680 K by reproducing the data points via the Brillouin function. Additionally, a zero-field cooled – field cooled (ZFC–FC) magnetization measurement spanning the entire relevant temperature range from 5 to 773 K can be found in Fig. S3.

### Magnetic phase transition via heat treatment

**Figure 3:**
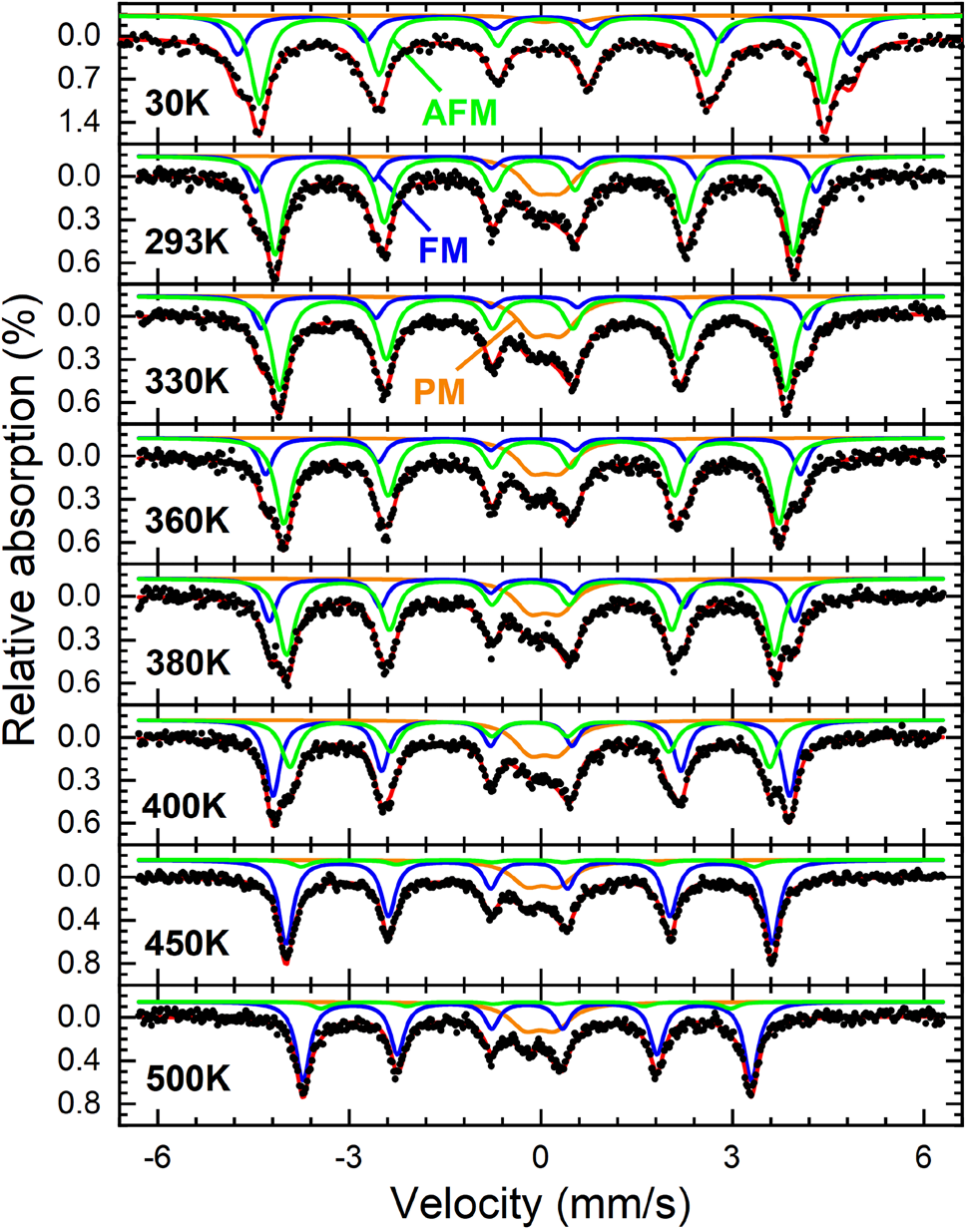
Mössbauer spectra of FeRh nanoparticles after annealing recorded at a temperature range of 30 – 500 K. Subspectra can be assigned to the low-temperature AFM state (green), the high-temperature FM state (blue), and an additional (super-) paramagnetic doublet contribution (orange).

**Figure 4:**
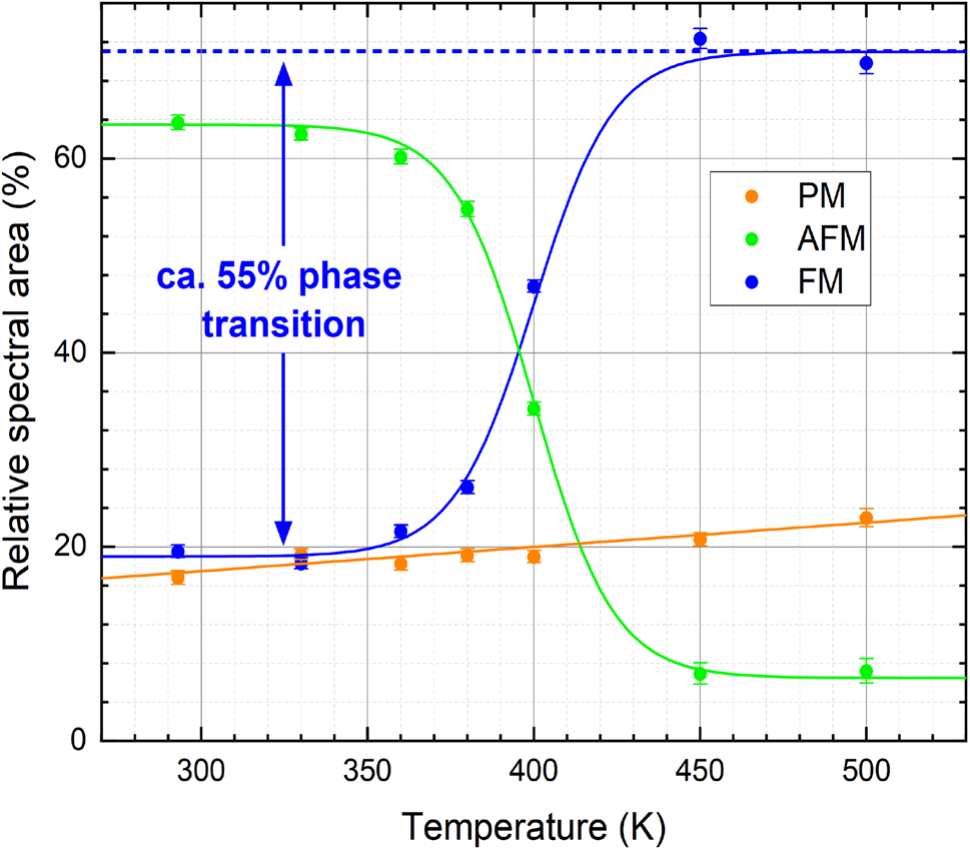
Relative spectral areas of individual contributions observed in the Mössbauer spectra of the annealed FeRh nanoparticles: (super-)paramagnetic doublet (orange), low-temperature AFM-state (green) and high-temperature FM-state (blue). Sigmoidal interpolation curves provide a guide to the eye. After the initial fitting of experimental Mössbauer spectra, hyperfine parameters B_HF_ and isomer shift δ were regulated by their known temperature-dependent behavior to ensure a higher precision in the determination of the shown subspectral areas.

In order to clarify the phase composition of the sample analyzed by magnetometry at different annealing temperatures and to relate the magnetization results to the FeRh paramagnetic (PM), AFM, and FM phases, Mössbauer spectroscopy measurements are performed. The evaluated temperature range is 30 – 500 K, focussing on the AFM-FM transition at 300 – 450 K, as shown in Fig. 3. The data indicates a superposition of two sextet subspectra and a minor broadened structure (orange) at the center of the spectra. Due to the low absorption, its fine structure cannot be resolved entirely, whereby it has been reproduced via a broadened doublet subspectrum. This component is assigned to the PM γ-FeRh state, possibly overlapping with a minor superparamagnetic contribution of the smallest nanoparticles, while the two sextet subspectra represent the AFM low-temperature (green) and FM high-temperature state (blue). This assignment has been verified by comparison of the hyperfine parameters to those of epitaxial FeRh films, including the slightly higher hyperfine magnetic field B_HF_ of the ferromagnetic contribution^44^. At ca. 400 K, the phase transition to the FM-state is most prominent, as be seen by the reversal in subspectral intensity. This is more noticeable in Fig. 4, showing the relative spectral intensities, revealing that ca. 50—60% of the Fe atoms undergo the phase transition. However, it should be noted that even at temperatures below the phase transition, a minor FM-fraction is visible, while a minuscule spectral intensity of lower B_HF_, usually representing the AFM state, remains even up to 500 K. This could indicate either that a distinct broadening of the FeRh thermal hysteresis in the nanoparticles is occurring, as it has been reported e.g. for thin FeRh films^64^, or that a fraction of nanoparticles does not exhibit the phase transition, as the FM state is stabilized due to structural disorder or variations in the local stoichiometry^44^.

**Figure 5:**
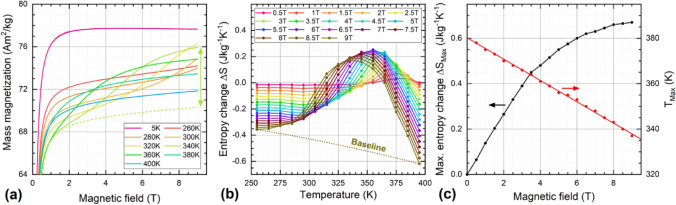
(**a**) Field-dependent magnetization curves recorded at 5—400 K in magnetic fields up to 9 T showing the field-induced AFM-FM phase transition, the dashed line is a guide to the eye extrapolating the curve at 340 K in the absence of the phase transition, (**b**) entropy change ΔS extracted from (**a**), the dotted line represents a background component related to the temperature-dependent decrease in high-field magnetization, (**c**) maximum entropy change ΔS_MAX_ (black), and shift in maximum position T_Max_ (red).

After observing the FeRh thermal hysteresis (Fig. 2a) for the annealed samples, the magnetocaloric properties are studied in more detail. To do so, the field-dependent magnetization curves were recorded up to 9 T at temperatures increasing from 5 K up to 400 K, Fig. 5a. As the initially annealed material had been mixed with boron nitride for Mössbauer spectroscopy measurements, a new sample from the same batch was prepared following the same annealing procedure, yielding slightly lower high-field magnetization. Although in these measurements a state of high magnetization already seems evident even at low temperatures, a field-driven transition to the full FM-state is visible as an additional sigmoidal increase in magnetization ∆M. At the maximum attainable fields, this effect already takes place at ca. 280 K, indicating a broadened thermal hysteresis in this sample. In contrast, the field-induced magnetization increase is most prominent at ca. 340 K. The expected M(H) trend in absence of field-induced effects is shown as a comparison (dashed green line), from which ∆M can be estimated to be ca. 6 Am^2^/kg (arrow). For higher temperatures, this increase shifts to lower fields, which is a well-known effect in FeRh^65^. Above ca. 400 K, the sample is already ferromagnetic even in the absence of considerable magnetic fields.

Figure 5b shows the entropy change ∆S close to the phase transition temperature, calculated from data in Fig. 5a following the approach proposed by Percharsky et al^66^. It should be taken into account that compared to the saturation magnetization, the field-driven increase in ∆M is relatively moderate and the phase transition of the FeRh nanoparticles is strongly broadened. Therefore, the peak in ∆S corresponding to the phase transition is superimposed by a strong background signal originating from the slow decrease in M_S_(T) upon rising temperature, following the Brillouin function. Figure 5c specifies the magnetic field dependence of the maximum entropy change ∆S_Max_. As expected for FeRh the phase transition shifts by about 4–5 K/T. ∆S_Max_ begins to saturate at the maximum attainable field, close to ∆S_Max_(9 T) ≈ 0.7 Jkg^-1^ K^-1^.

Based on the data shown in Fig. 5, it is evident that the field-induced increase in magnetization is rather limited, while a high-magnetization state is already attainable at lower fields or temperatures, respectively, without crossing the phase transition temperature. It is not completely clear why the moderate increase in high-field magnetization seems to correspond to app. 55% of Fe-atoms being involved in the phase transition as demonstrated in Mössbauer experiments. As mentioned before, this is likely connected to a stabilization of parts of the nanoparticle volume in the FM-state due to structural disorder potentially in conjunction with local variations in stoichiometry. Also, minor differences in B2-phase fraction in the two samples used for Mössbauer spectroscopy and magnetocaloric analysis are possible, despite similar preparation, affecting the observed magnetization increase.

**Figure 6:**
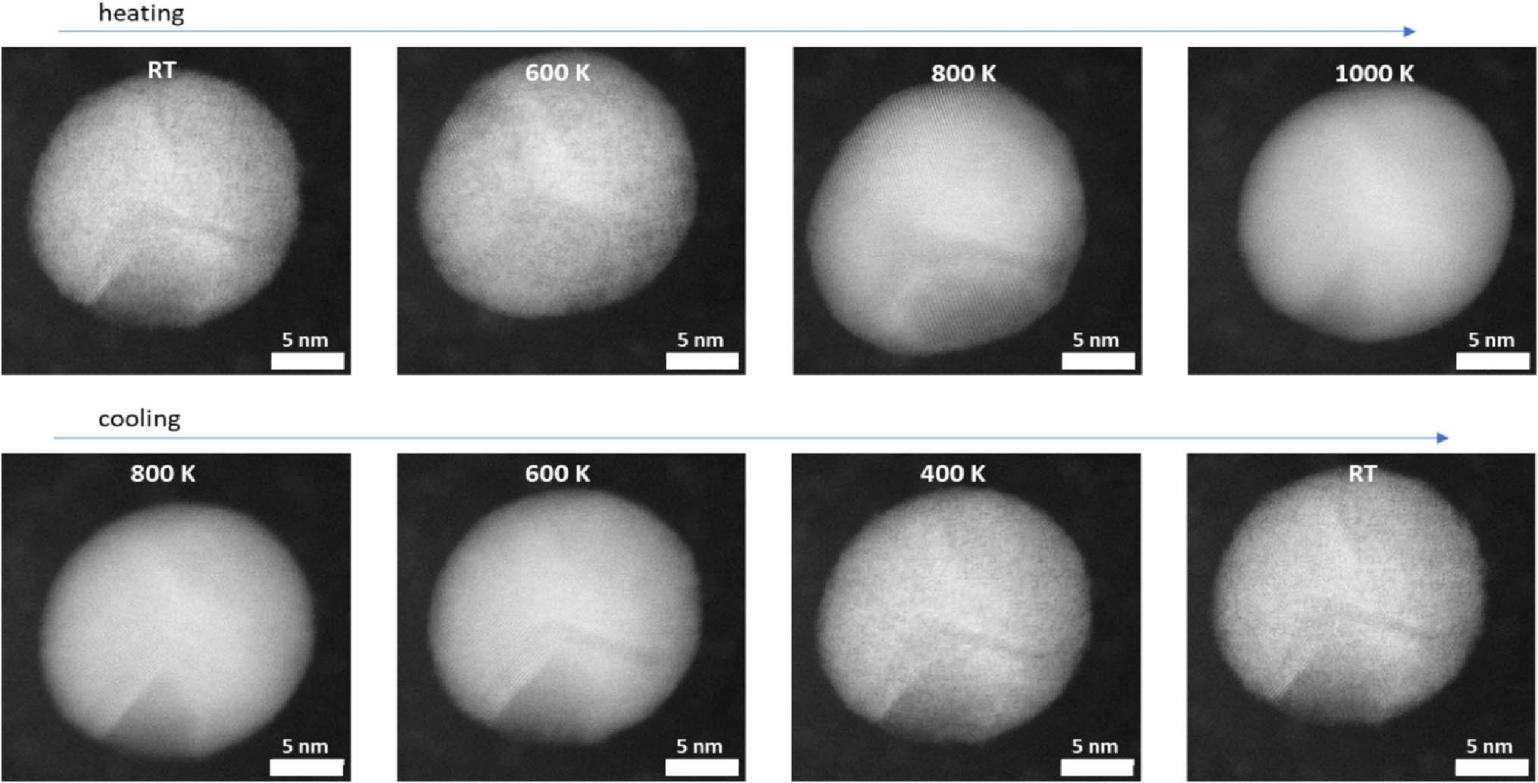
Dark-field TEM images of a selected FeRh nanoparticle evolution for different in-situ annealing temperatures.

High-temperature TEM measurements were performed as a control experiment (Fig. 6) to exclude a change in nanoparticle size or shape as a possible origin for the increase in magnetization. As seen in Fig. 2, the magnetization increases with the annealing temperature. In addition to the phase change, structural changes such as phase separation, core–shell formation, or growth of the particles can also influence the magnetization. To consider, a particle was heated in-situ up to 1000 K and cooled down again. At the beginning, the particle had darker areas, which could represent a different crystal structure or a non-uniform element distribution. However, even after heating, the particle remains unaltered, pointing out that the nanoparticles do not experience elemental segregation due to the heating. Besides, from the dark-field TEM images, a d-spacing of 0.23 ± 0.01 nm is obtained for the FeRh nanoparticle. It should be noted that the FeRh nanoparticle shows no apparent difference in the morphology and d-spacing with increasing temperature, as observed in the TEM images (Fig. 6).

### Laser sintering

**Figure 7:**
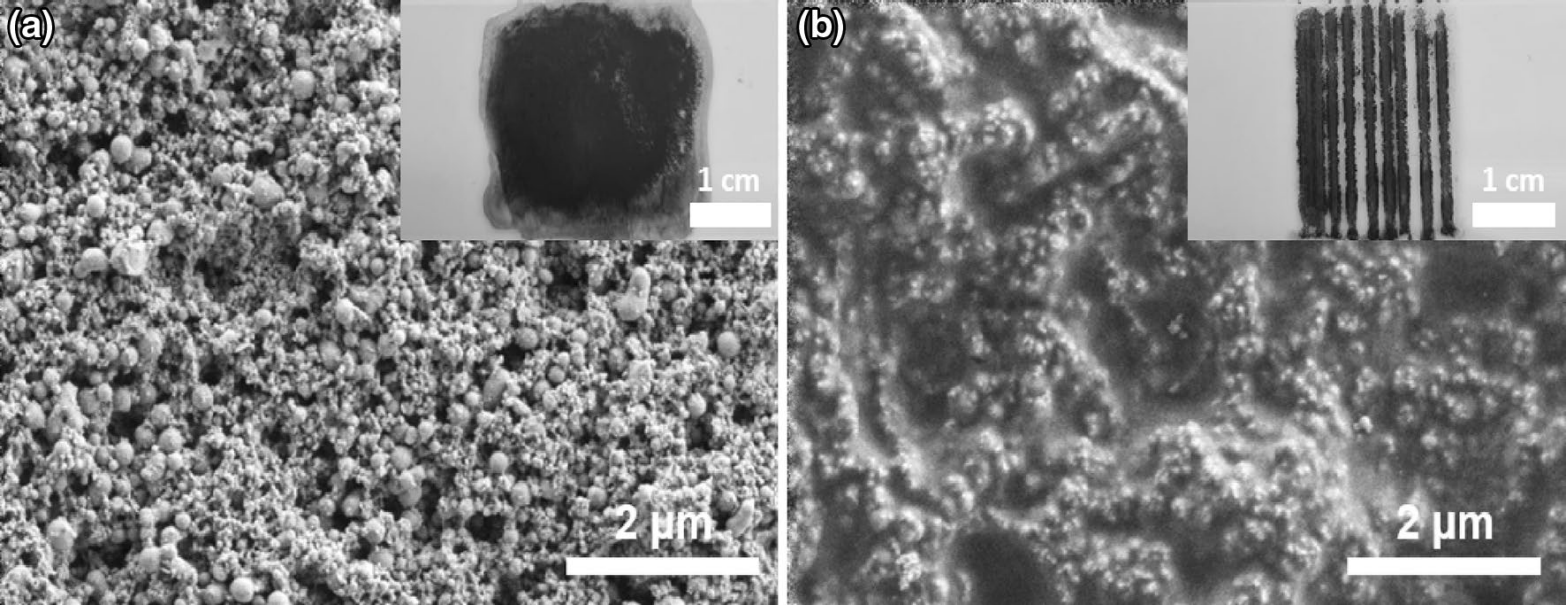
SEM image with respective light optical microscopy image of the drop-casted FeRh ethanol-based ink on a glass substrate (**a**) before and (**b**) after sintering with a CW-laser (0.85 W/mm^2^).

To demonstrate the feasibility of printing 2D structures, FeRh lines are sintered on a glass substrate. The ink dispersed on the substrate and the sintered lines are examined by scanning electron microscopy (SEM) (Fig. 7). The as-deposited nanoparticle ink shows a porous structure and consists of particles with a number-weighted mean diameter of 28 ± 18 nm. After laser sintering, the nanoparticles are densely compacted forming a bulk structure with almost no observable pores. However, at the surface, the single particles can still be distinguished (Fig. 7b). The success of the sintering was also confirmed by measuring the sheet resistance of the sintered lines and comparing it to the initially deposited ink using a 4-point probe device. The sheet resistance is an indicator of the electrical resistance of the material. Since the electrical resistance depends on the interparticle distance, porosity, and compactness of the sample, the better the consolidation of particles into bulk structures, the lower the sheet resistance^67^. The sheet resistance of the as-prepared FeRh thin film could not be measured, as the values were higher than the detection limit of the equipment (10 MΩ/sq). This film was prepared by direct drop casting of the FeRh ink on a glass substrate to avoid any influence of the PVP layer that would strongly reduce the electrical conductivity. The measured mean sheet resistance of the sintered FeRh lines was 81.3 kΩ/sq. This shows that the polymer is evaporated due to the laser irradiation, allowing the sintering of the nanoparticles into compact structures, improving the electrical conductivity.

**Figure 8:**
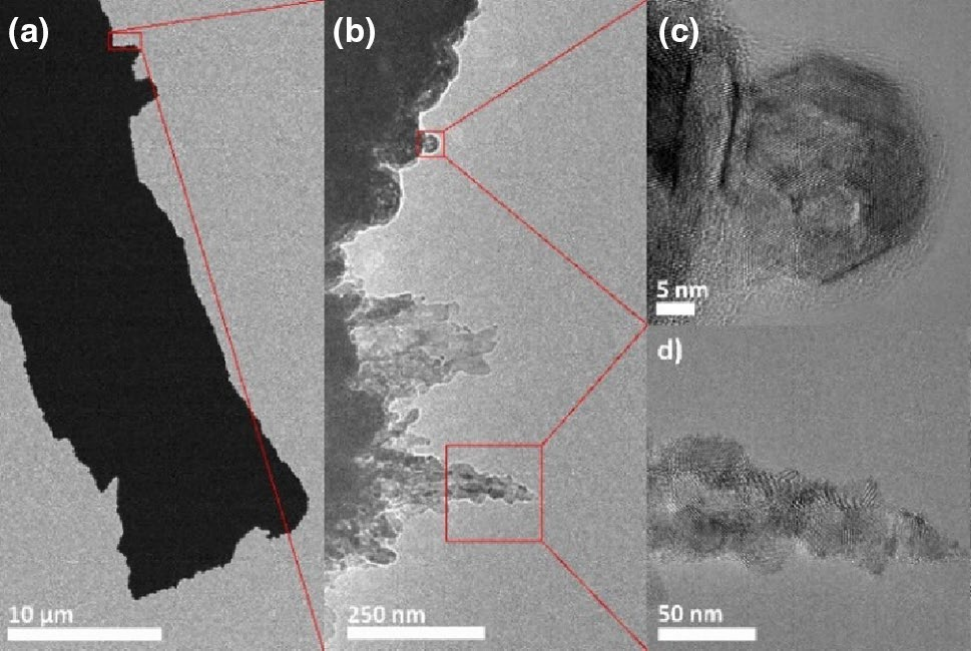
Bright-field TEM micrographs of laser-sintered FeRh. (**a**) Overview of laser-sintered flake. (**b**) Bright-field image showing spherical particles and dendritic formations. (**c**) HR-TEM of a single spherical particle, sintered to the surface of the flake. (Compare Suppl. Fig. S5) (**d**) HR-TEM of a dendrite composed of multiple crystallites.

Additionally, laser sintering was performed on an electron transparent membrane, creating a free-standing flake (Fig. 8a) of laser-sintered FeRh structures for TEM investigations. Along the perimeter of this flake, we observed areas with spherical particles and regions with dendritic structures (Fig. 8b). High-resolution images show that the spheres are single-crystal objects sintered to the flake's surface (Fig. 8c). In contrast, the dendrites (Fig. 8d) are composed of multiple crystallites embedded in an amorphous matrix. Since no amorphous phase was seen in the TEM analyses of the particles before sintering, the amorphous phase is a potential residue of PVP or SiN from the destroyed substrate after laser sintering. Both fcc and bcc structures are observed in the sintered flake (compare Supplementary Fig. S5). However, the morphological features observed in TEM may be influenced by the fact that there is no heat sink under the membrane. The thermal distribution during laser pulses may thus be slightly different from that on thicker substrates.

**Figure 9:**
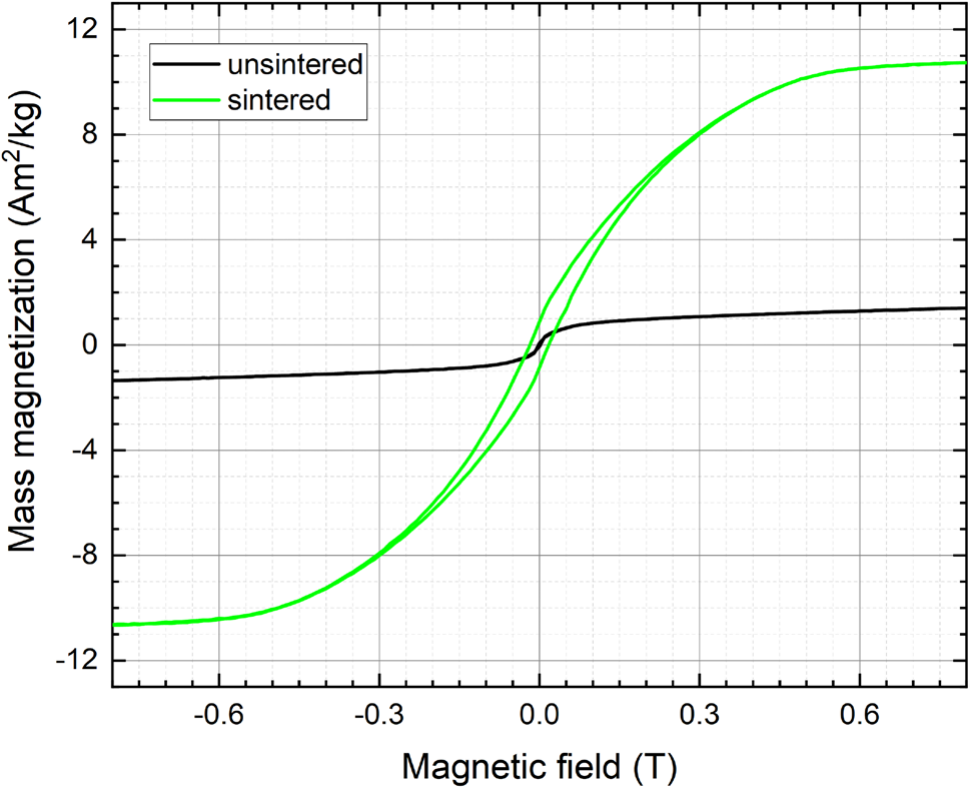
Magnetic hysteresis loops of the FeRh nanoparticle ink recorded before and after laser sintering (6 W), after subtraction of the diamagnetic glass-substrate signal.

The purpose of laser sintering was not only to demonstrate the possibility of achieving 2D printing of the generated nanoparticles but also to induce magnetization by triggering a phase change from γ-FeRh to B2-phase. In order to characterize this possible phase change, magnetometry measurements were conducted before and after laser sintering of the nanoparticle ink (Fig. 9). While the unsintered material after background subtraction displays only a minimum magnetization and no coercive field, the sample after laser sintering exhibits an enhanced magnetization of M(0.8 T,300 K) ≈ 11 Am^2^/kg and visible magnetic hysteresis, indicating a partial transformation to the B2-phase despite the very short heating time during laser irradiation, which is in agreement with the TEM findings. A direct comparison of the sintered lines’ magnetization value with the nanoparticle magnetization obtained at different annealing temperatures (Fig. 2c) yields an equivalent long-time heating temperature of app. 600 K is achieved by laser sintering. The lower equivalent processing temperature during laser sintering compared to the optimum nanoparticle annealing at 973 K explains the lower increase in magnetization of the laser-sintered sample when evaluated against the maximum nanoparticle magnetization at 300 K after annealing at 973 K (M(0.8 T,300 K) = 96 Am2/kg). Upon future optimization of laser sintering parameters, a further increase in the magnetization and the associated B2-phase fraction, as well as magnetocaloric performance, is expected.

## Conclusion

We conclude that the metastable γ-Fe_50_Rh_50_ nanoparticles produced by laser ablation in liquid can be processed into the stable B2-phase by annealing. XRD and magnetometry results show the beginning of a transformation from the γ-phase to the B2-phase at a temperature of ca. 550 K, whereas at around 723 K the characteristic FeRh thermal hysteresis starts to develop. At 1000 K, 90% of the nanoparticles are in the B2-state. Temperature-dependent magnetization measurements indicate a broadened AFM/FM transition, especially upon cooling to the AFM state, while M(H) curves recorded upon increasing sample temperature verified the field-induced transition to the FM-state, increasing the magnetization by an additional ~ 10%. Surprisingly, Mössbauer measurements display that about 50% of the annealed sample undergo the Fe_50_Rh_50_ characteristic AFM-FM transition, with the inflection point of the phase transition detectable at about 400 K. This apparent contradiction of the material fraction involved in the phase transition possibly originates from structural disorder and spin frustration. High-temperature TEM measurements confirm that there are no morphological changes or elemental segregation. The heating experiments were effectively transferred to the laser sintering process showing the potential for 2D-printing. The conversion to B2-FeRh after laser sintering was indicated by TEM as well as by the magnetization increase measured, showing the potential of our method to produce magnetocaloric structures.

## Supplementary Information

Supplementary figures.
