# Publisher Correction: Towards laser printing of magnetocaloric structures by inducing a magnetic phase transition in iron-rhodium nanoparticles

**DOI:** 10.1038/s41598-021-96847-x

**Published:** 2021-08-27

**Authors:** Ruksan Nadarajah, Joachim Landers, Soma Salamon, David Koch, Shabbir Tahir, Carlos Doñate‑Buendía, Benjamin Zingsem, Rafal E. Dunin‑Borkowski, Wolfgang Donner, Michael Farle, Heiko Wende, Bilal Gökce

**Affiliations:** 1 Universitätsstr. 7, 45141 Essen, Germany; 2 Faculty of Physics and Center for Nanointegration Duisburg‑Essen (CENIDE), University of Duisburg-Essen, Lotharstr. 1, 47057 Duisburg, Germany; 3 Institute of Materials Science, Technical University of Darmstadt, Alarich‑Weiss‑Strasse 2, 64287 Darmstadt, Germany; 4 Materials Science and Additive Manufacturing, University of Wuppertal, Gaußstr. 20, 42119 Wuppertal, Germany; 5 Ernst Ruska-Centre for Microscopy and Spectroscopy With Electrons and Peter Grünberg Institute, Forschungszentrum Jülich GmbH, 52425 Jülich, Germany

Correction to: *Scientific Reports* 10.1038/s41598-021-92760-5, published online 02 July 2021

The original version of this Article contained an error where Figures 3 and 4 were interchanged. The original Figures 3 and 4 and accompanying legends appear below.

**Figure 3:**
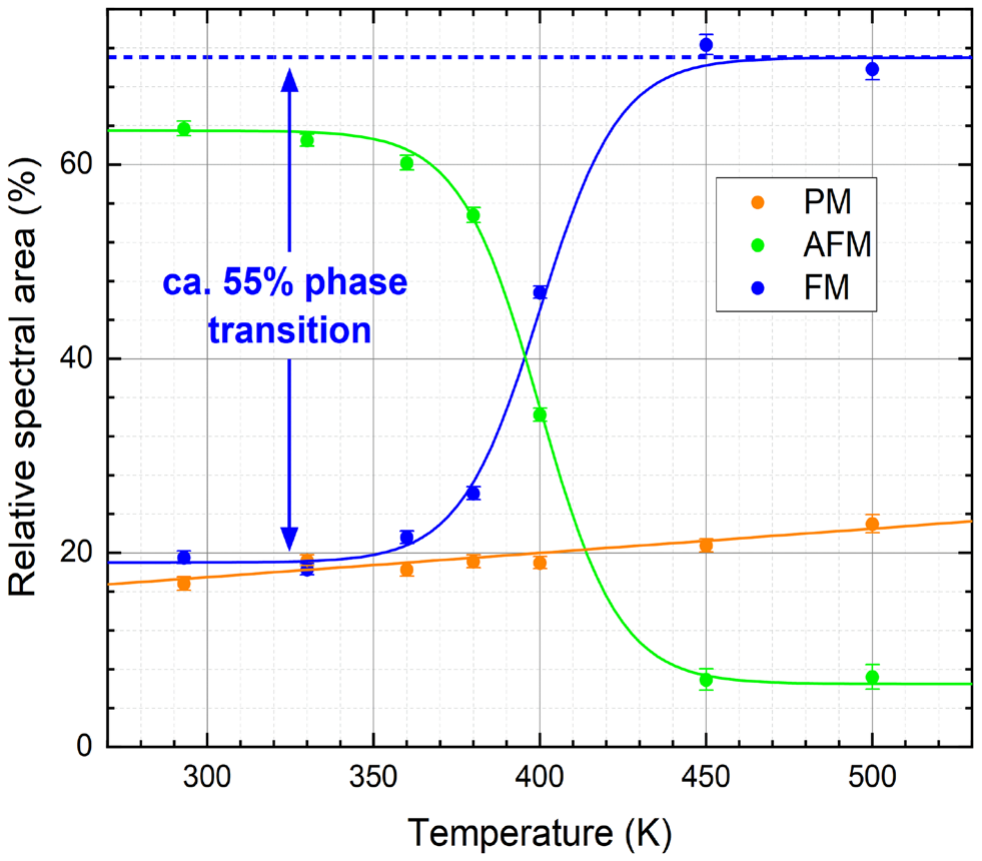
Mössbauer spectra of FeRh nanoparticles after annealing recorded at a temperature range of 30 – 500 K. Subspectra can be assigned to the low-temperature AFM state (green), the high-temperature FM state (blue), and an additional (super-) paramagnetic doublet contribution (orange).

**Figure 4:**
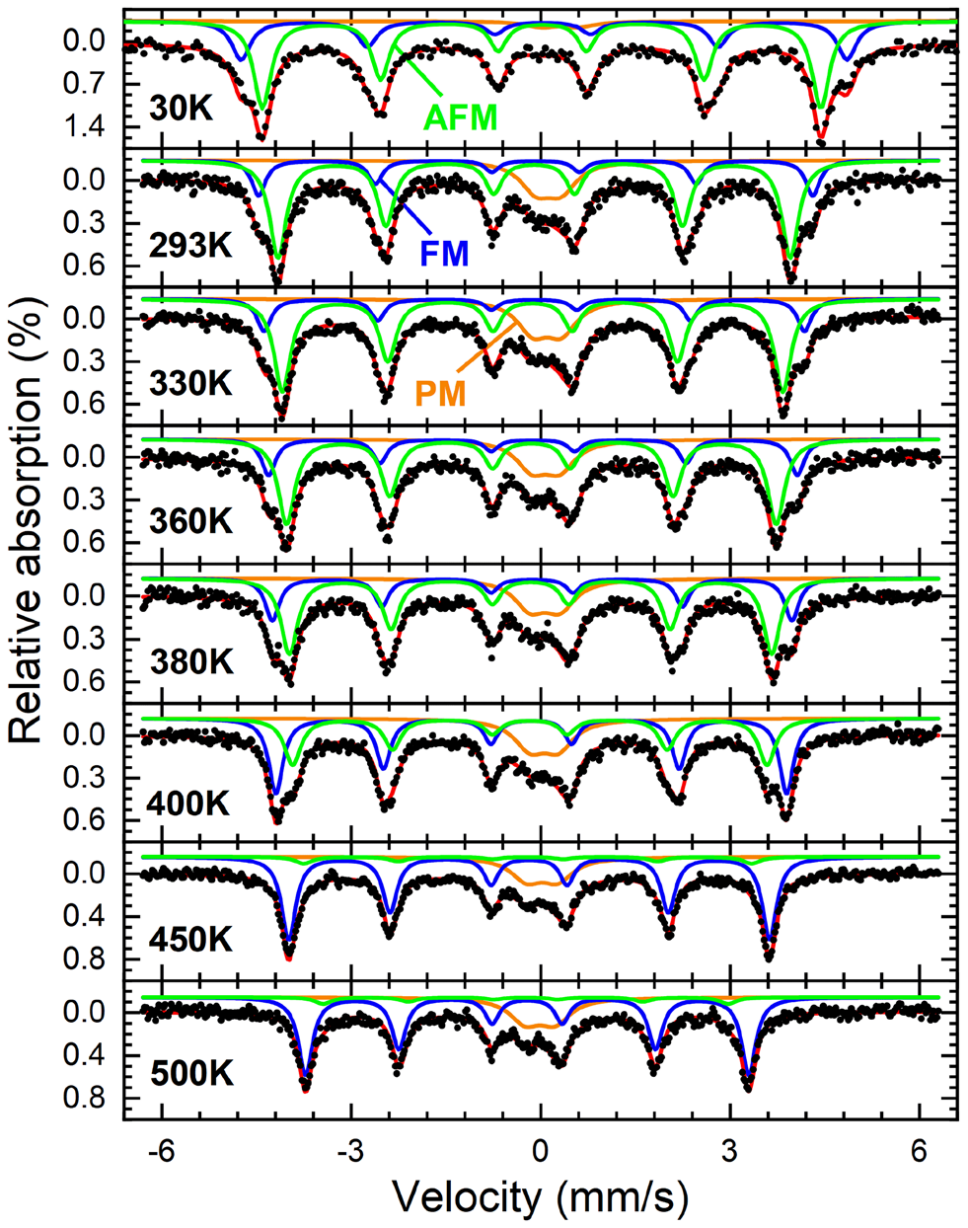
Relative spectral areas of individual contributions observed in the Mössbauer spectra of the annealed FeRh nanoparticles: (super-)paramagnetic doublet (orange), low-temperature AFM-state (green) and high-temperature FM-state (blue). Sigmoidal interpolation curves provide a guide to the eye. After the initial fitting of experimental Mössbauer spectra, hyperfine parameters B_HF_ and isomer shift δ were regulated by their known temperature-dependent behavior to ensure a higher precision in the determination of the shown subspectral areas.

The original Article has been corrected.

