# Supplementary material for: Towards laser printing of magnetocaloric structures by inducing a magnetic phase transition in iron-rhodium nanoparticles
Source: Sci Rep. 2021 Jul 2;11:13719. doi: 10.1038/s41598-021-92760-5 (PMC8253782; doi:10.1038/s41598-021-92760-5)
Supplement: Supplementary file 1 — Supplementary figures. [file 41598_2021_92760_MOESM1_ESM.docx]

**Supplementary Information:** **Towards laser printing of magnetocaloric structures by inducing magnetic phase transition of iron‑rhodium nanoparticles**

Figure S1: B2 phase fraction depending on the annealing time @700 K.


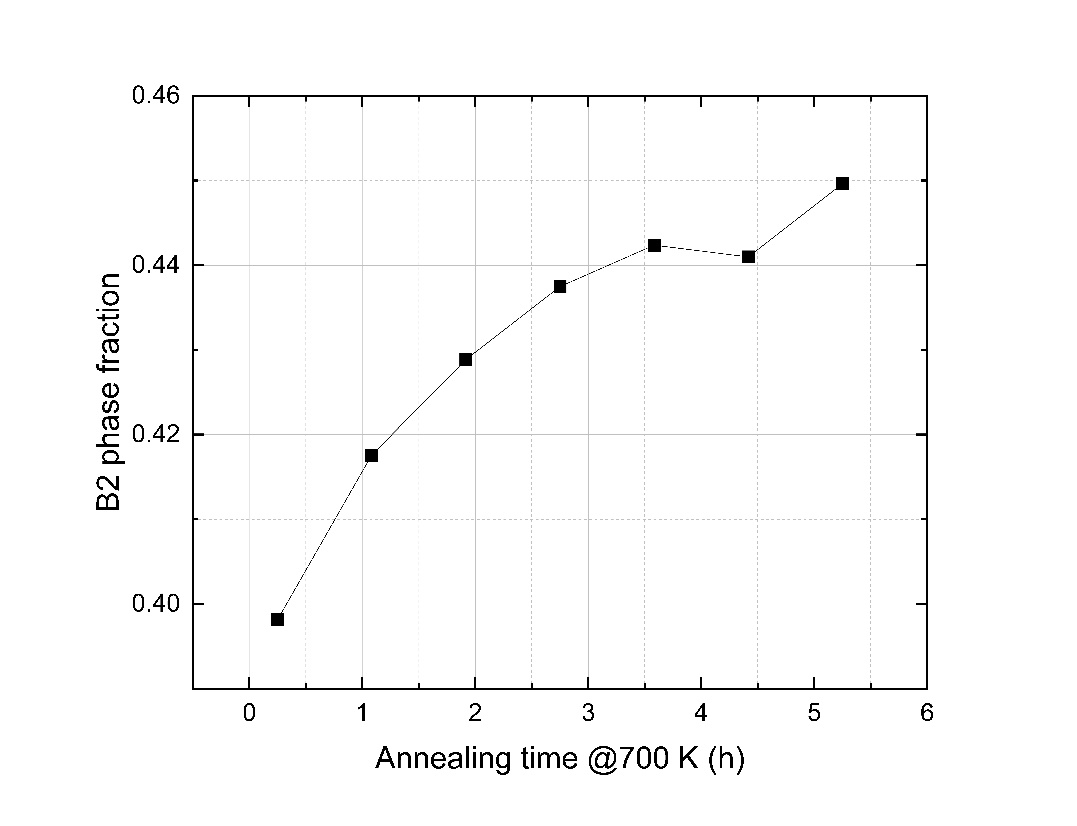


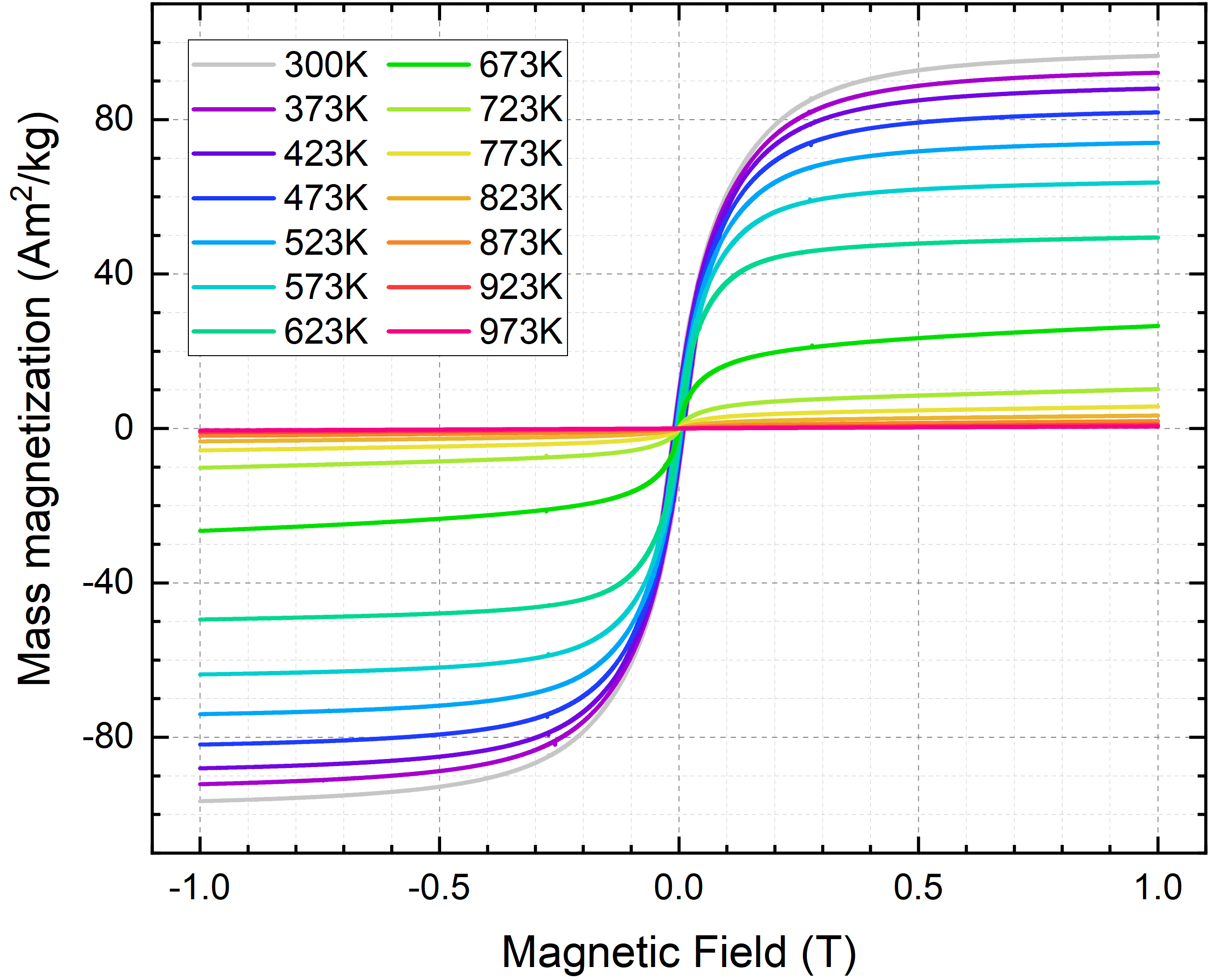


Figure S2: M(H)-curves of FeRh nanoparticles recorded at the respective annealing temperature T_H_ after completion of the measurement series displayed in figure 2.


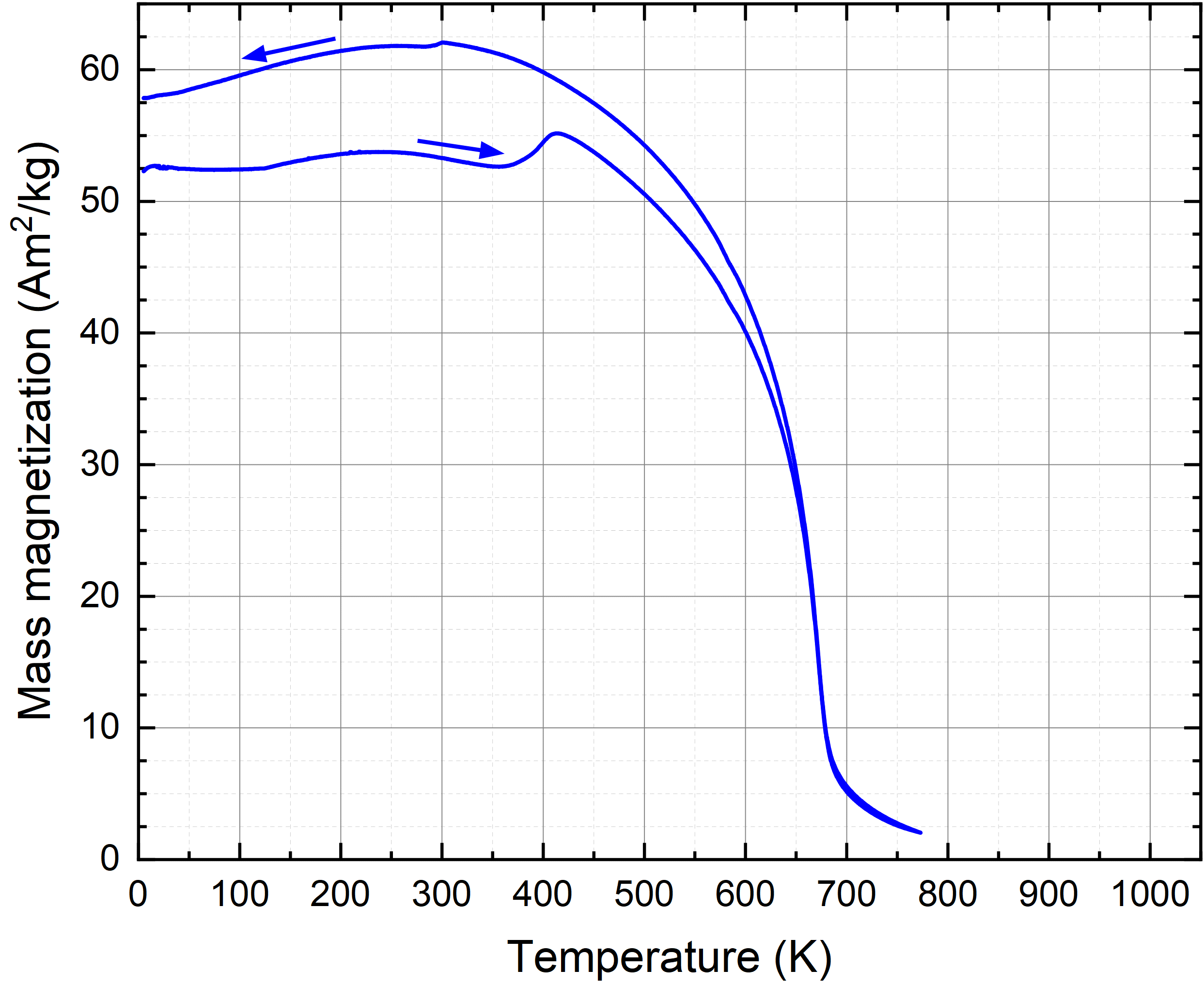


Figure S3: ZFC-FC magnetization measurement of the annealed FeRh nanoparticles recorded at 0.1T between 5 K and 773 K.

Figure S3 shows a ZFC-FC magnetization measurement in the complete range of 5-773 K recorded in an external magnetic field of 0.1 T for the sample state after annealing. A small fraction of the sample displays the characteristic FeRh AFM to FM phase transition ca. at 390 K, while a high fraction of the sample material already exhibits ferromagnetic properties (high magnetization) independent of the temperature. After cooling the sample material from above T_C_, the transition back to the low temperature state is strongly smeared out, indicating a very broad range of transition temperatures compared to bulk-FeRh, which could be explained by the minor AFM state to be metastable and reachable only by slow cooling to very low temperatures. This would be in agreement to the small residual FM fraction present in Mössbauer spectra even well below 360-400 K.

Figure S4: Magnetization depending on the B2 phase fraction.


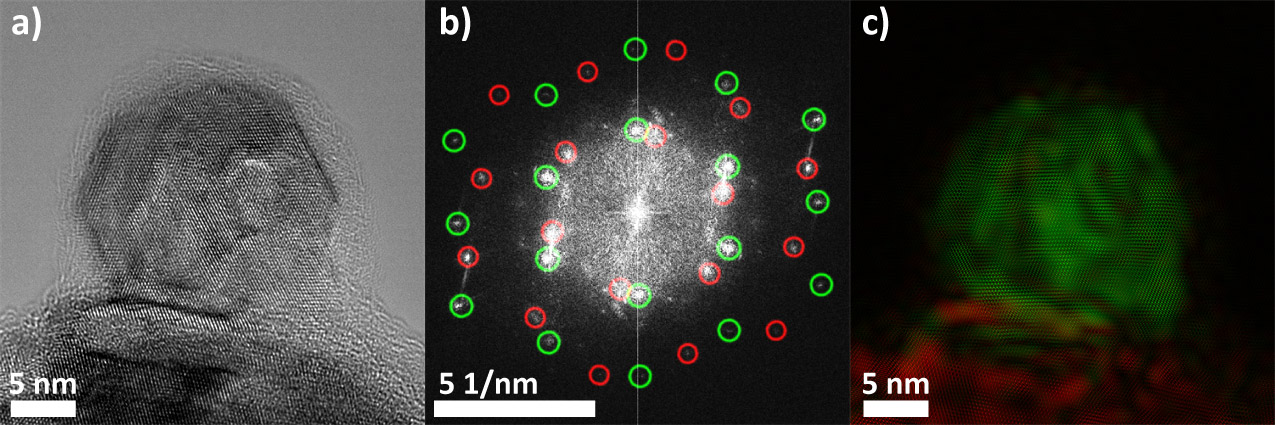


Figure S5: HR-TEM of sintered NP on FeRh Flake. a) HR-TEM micrograph, b) FFT of a). Red circles highlight indices corresponding to a bcc crystal in {012} orientation. Green circles highlight indices corresponding to an fcc crystal with its {114} orientation parallel to the beam axis. c) Inverse FFT of bcc indices and fcc indices combined into a false color image. The green color channel of this RGB image contains only the inverse FFT of the spots highlighted green in b. The Red color channel, the inverse of red highlighted spots, correspondingly.
